# The Atlanta Medical Library Association

**Published:** 1908-02

**Authors:** 


### EDITORIAL NOTES AND COMMENTS.

The Business Office of the JOURNAL-RECORD is 1 1-2 to 5 1-2 South Broad Street.

The Editorial Office is 318-319-320 The Prudential.

Address all Business Communications to Dr. George Brown, Manager.

Make remittances payable to THE JOURNAL-RECORD OF MEDICINE.

On matters pertaining to the Editorial and Original communications, address Dr. Bernard Wolff, Atlanta.

Reprints of original articles will be furnished at cost price. Requests for the same should always be made on THE MANUSCRIPT.

We will present, postpaid, on request, to each contributor of an original article, twenty (20) marked copies of THE JOURNAL-RECORD OF MEDICINE containing such article

### THE ATLANTA MEDICAL LIBRARY ASSOCIATION.

The Atlanta Medical Library Association is an institution which was established years ago, and, after a long period of suspended animation, was resuscitated four or five years ago and has been conducted with fair success since that time.

A portion of the Carnegie Library is set aside for its possessions in the matter of books and bound and current periodicals.

In the last named are most of the important medical journals general and special, published in this country, England and Germany.

It has been the desire of those most closely concerned in the library, to effect some sort of junction with the Fulton County Medical Society, and, through its protection and support, to secure not only a permanent place of deposit for the books, but a meeting place for the Society. Movement in this direction is under way and it is thought that an agreement will be reached in the near future.

The purpose is the purchase of a lot upon which a suitable building may be erected, funds for the latter being expected from certain philanthropic sources.

It is hoped that the medical profession of this city will unite in carrying forward to fruition this very laudable enterprise. The value of a well equipped reference library of medicine and a full list of medical journals is incalculable.

Work of a high character, without great personal expense, can not be prosecuted without such assistance, and without such work the medical profession will soon fall out in the march of progress.

